# Advances in Plants-Derived Bioactives for Cancer Treatment

**DOI:** 10.3390/cells12081112

**Published:** 2023-04-08

**Authors:** Natália Cruz-Martins

**Affiliations:** 1 Faculty of Medicine, University of Porto, 4099-002 Porto, Portugal; ncmartins@med.up.pt or ncmartins@ipsn.cespu.pt; 2 Institute for Research and Innovation in Health (i3S), University of Porto, 4099-002 Porto, Portugal; 3 Institute of Research and Advanced Training in Health Sciences and Technologies, CESPU, Rua Central de Gandra, 1317, 4585-116 Gandra, Portugal; 4 TOXRUN—Toxicology Research Unit, University Institute of Health Sciences, CESPU, CRL, 4585-116 Gandra, Portugal

Cancer, while a multifactorial chronic disease with an increasing prevalence, has been the subject of intense investigation, not only because of the growing need to find the main triggers that motivate its onset but essentially because of the need to discover increasingly safer and effective therapeutic options that have fewer adverse effects and associated toxicity. Of particular interest are the advances stated in targeted therapy, which have pronouncedly contributed to the amelioration of quality of life and to improve the survival of individuals living with the disease. However, adverse effects and acquired resistance resulting from the occurrence of genetic mutations have limited its effectiveness, and therefore new strategies have been searched with the intent to overcome the current limitations. Among the various strategies, the use of new drug delivery systems, namely nanoformulations, stands out as a way of improving the effectiveness of conventional treatments and reducing their adverse effects.

However, in this context, more and more attempts have been conducted to create new therapeutic formulas combining conventional drugs with natural bioactives. Used since immemorial times, medicinal plants are composed of a broad pool of bioactive molecules with multiple biological effects that have been intensely studied in recent years for various purposes, including in the field of oncology. The present Special Issue, entitled “Advances in Plants-Derived Bioactives for Cancer Treatment”, intends to clarify the latest advances stated in oncology by studying of effects of natural bioactives derived from plants, whether of natural or synthetic origin, used alone or in combination with conventional drugs to improve their overall efficacy and reduce adverse effects.

In this Special Issue, a total of 18 works were published, including 12 original investigations and 6 review articles. Regarding the types of cancer studied, breast, followed by colorectal, intrahepatic cholangiocarcinoma, glioblastoma, mesothelioma, chronic myeloid leukemia, lung, pancreatic and head and neck squamous cell carcinoma are those addressed in the papers published in this Special Issue. Despite all the studies being of a preclinical nature, mainly in vitro and in silico studies, the findings stated here are of the utmost importance to boost new advances in this area and contribute to the design of increasingly detailed studies.

Looking at the original works published, and starting with one of the most common cancers worldwide, breast cancer, Arora et al. [1] addressed, from a preventive perspective, the ability of broccoli sprouts and green tea polyphenols to prevent mammary tumors at early stages through genome-wide analysis on methylome and transcriptome. As main findings, the authors stated that genes were differentially expressed and methylated in response to these botanicals, being, however, more efficient when used in combination. Thus, the use of these botanicals in combination was revealed to be more effective in preventing and inhibiting breast cancer by interfering with key tumor-related genes. In another study, Genovese et al. [2] addressed the anticancer activity of *Orobanche crenata* Forssk. leaf extract in human breast cancer cell lines, where there was stated a dose-dependent inhibitory activity. Park et al. [3] also addressed the ability of natural compounds to selectively target genes regulated by extracellular acidosis. Among the 102 compounds evaluated in silico, bruceine D was revealed to have the highest therapeutic potential, being capable of regulating reprogrammed genes driven by acidosis while also modulating the tumor cell–TME interactions and reducing the amyloid beta precursor protein and CD44 expression, ultimately improving the overall survival of patients living with the disease [3].

Colorectal cancer is another increasingly searched-for cancer type, owing to its raising incidence, associated mortality, and poor prognosis, in addition to the fact that it is often discovered at advanced stages. Barboura et al. [4] assessed the ability of tannic acid to counteract the transforming growth factor-b-induced epithelial-mesenchymal transition in human and murine colorectal cancer cell lines. In this work, among other aspects, tannic acid was able to reverse the epithelial-mesenchymal transition through repressing mesenchymal and re-expressing epithelial markers, possibly resulting from the disruption of the non-canonical signaling pathway induced by transforming growth factor β1 (TGF-β1). Additionally, it is noteworthy that in vivo tannic acid was capable of significantly delaying tumor growth without exerting liver and kidney toxicity. In the same line, Bakshi et al. [5] addressed the ability of crocin to inhibit angiogenesis and metastasis in colon cancer cell lines. As their main findings, the authors stated that crocin markedly decreased the viability of cancer cell lines exerting any toxicity in normal counterparts, mostly through the inhibition of cell migration, invasion, and tube formation in a dose-dependent fashion. Concomitantly, these findings were observed in vivo and stated that crocin inhibited angiogenesis and colorectal cancer metastasis through the modulation of nuclear factor κappa B (NF-κB) and blockage of tumor necrosis factor α (TNF-α)/NF-KB/VEGF pathways [5].

The work of Tomooka et al. [6] addressed the ability of sulforaphane to potentiate the anticancer effects of gemcitabine on human cells of intrahepatic cholangiocarcinoma. As their main findings, sulforaphane was capable of intervening at the level of the cell cycle and cell invasion and decreased the expression of pro-angiogenic markers, and thus such a combination therapy provides a promissory option for the treatment of this cancer.

Glioblastoma is another life-threatening chronic condition with a raising healthcare burden. Majchrzak-Celińska et al. [7] addressed the ability of anticancer effects and this mechanism of action on six lichen secondary metabolites in glioblastoma cell lines. As the main findings, these compounds, namely atranorin, caperatic, physodic, squamatic, salazinic, and lecanoric acids, were capable of crossing the blood–brain barrier and generating cytotoxicity to cancer cells lines, mainly through generating oxidative stress, interfering with the cell cycle, inducing apoptosis and inhibiting the Wnt-b-catenin pathway, making these effects stronger when temozolomide was used in combination [7].

The work by Cuciniello et al. [8] addressed the anticancer effects of aglianico grape seed semi-polar extract in mesothelioma cell lines. Specifically, in this work, the authors stated, through transcriptomic and metabolomic analyses, that the extract exerted a pronounced pro-apoptotic effect by modulating murine double minute 2 (MDM2) expression and ultimately inhibiting tumor progression.

In another study, the anticancer activity of a natural compound, in this case, daphnoretin, was tested by Huang et al. [9] in human chronic myeloid leukemia cells. As their main finding, the authors observed that daphnoretin intervened at the level of the cell cycle, inhibiting tumor growth and also inducing megakaryocytic differentiation. In the same line, Sitarek et al. [10] assessed the anticancer activity of diterpenes isolated from *Plectranthus ornatus* Codd. in leukemia and lung cancer cell lines. The compounds halimane and labdane diterpenes (11R*,13E)-11-acetoxyhalima-5,13-dien-15-oic acid and 1α,6β-diacetoxy-8α,13R*-epoxy-14-labden-11-one and the forskolin-like 1:1 mixtures of 1,6-di-*O*-acetylforskolin and 1,6-di-*O*-acetyl-9-deoxyforskolin led to a pronounced reduction in the cancer cells’ viability while preventing the exertion of cytotoxic effects to normal cells lines.

The ability of phytochemicals to counteract pancreatic cancer, an increasingly devasting type of cancer, was also investigated by Cykowiak et al. [11]. Briefly, the authors addressed the ability of several natural compounds to modulate Nrf2 and NF-κB signaling pathways, thus attenuating pancreatic cancer within in vitro and in vivo models. Among the various compounds tested, xanthohumol and phenetyl isothiocyanate were used in combination revealed and were revealed to be more effective than single compounds at decreasing both the canonical and non-canonical activation of Nrf2 and also at decreasing the activation of NF-κB and subsequently reducing cytosolic cyclooxygenase 2 (COX-2) and nuclear signal transducer and the activator of transcription 3 (STAT3) [11].

Finally, among the original works, Kumar et al. [12] addressed the ability of 7-epitaxol to induce apoptosis and autophagy in head and neck squamous cell carcinoma. Among other statements, 7-epitaxol was capable of suppressing, to a large extent, the cancer cells’ viability mostly through inducing cell cycle arrest. These effects were mostly triggered by the ability of the compound to induce cell death, alter the mitochondrial membrane potential, and trigger chromatin condensation, at the same time, which augmented the activation of caspases 3, 8, 9, and Poly(ADP-Ribose) Polymerase 1 (PARP), while suppressed p62 expression and reduced the phosphorylation of mitogen-activated protein kinase (ERK1/2) [12].

In addition to the recent findings presented above from original studies, in this Special Issue, there are six review articles summarizing the most recent literature on: (1) the molecular targets and mechanisms of action of salicylic acid in plants and humans [13]; (2) the multitarget potential of berberine while antineoplastic for antimetastatic agents in lung cancer [14]; (3) the anticancer potential of *Spatholobus suberectus* Dunn. [15]; (4) the anticancer potential of natural products in solid tumors [16]; (5) the anticancer potential of phytochemicals and their mechanisms of action [17]; and (6) the preventive and therapeutic potential of anthocyanidins/anthocyanins (ACDs/ACNs) in prostate cancer [18].

In the first review, Ding and colleagues [13], while providing a detailed analysis of the shared and molecular targets and effects of salicylic acid in plants and humans, underlined the vast diversity of targets, which include this binding to salicylic acid that characterizes the biological processes and activities linked with it. Nonetheless, as highlighted by the authors, it remains questionable whether, in addition to the already identified targets, there are others of equal importance and also how salicylic acid binding to these receptors interferes with their activities and biological processes, and also about the effect of salicylic acid in real conditions. Taken together, all these aspects underline the need for the design of comprehensive analysis and quantification of multiple targets of salicylic acid and associated pathways to develop better strategies to improve crop plants and provide better therapeutic options for human diseases.

In the other work, Achi and colleagues [14] carefully detailed the main mechanisms of action of berberine as an antineoplastic and antimetastatic agent, which was underlined by the authors, as a promissory agent for the treatment of cancer, particularly lung cancer, whether used alone or in combination with other drugs. Nonetheless, when providing the poor pharmacokinetics of berberine, it can be rapidly eliminated from the body, and thus, new formulations, for example, nano-based delivery systems, have been proposed to overcome this limitation. Another recent limitation is the need to administer probiotics to improve the berberine bioavailability and sodium caprate to improve the cellular permeability of the molecule. Taken together, all these aspects highlighted by the authors are of utmost importance to ensure proper use and to benefit from the multiple benefits of berberine in cancer [14].

In the third review, the authors underlined that *S. suberectus* was composed of a rich variety of phytochemicals, including flavonoids, chalcone, dihydroflavone, pterocarpan, and phenolic acid to whom both in vitro and in vivo experiments underlined their anticancer effects. This plant exerts anticancer effects mainly through the modulation of PI3K/Akt/mTOR and Ras/Raf/MAPK pathways, which are, however, necessary to perform clinical studies and solidly conclude their therapeutic potential in humans [15].

In the review by Muhammad and colleagues [16], the authors carefully addressed the various signaling pathways modulated by phytochemicals, with particular emphasis on the multitarget potential of natural products, also underlining the need to design increasingly detailed studies to understand the mechanistic aspects related to their use as chemopreventive agents, as well as the need to design clinical studies that address their effectiveness in real clinical conditions.

In the fifth review, Khan and their colleagues [17] devoted particular attention to the anticancer potential of curcumin, epigallocatechin gallate, genistein, lycopene, and resveratrol, detailing their mechanisms of action and clinical findings, which have been stated, to date, particularly in breast and lung cancers, while also underlining the need to develop increasingly deepened studies to discover the real potential of plants and promote them as a source of new, safer, cheaper and more effective molecules to treat cancer than the currently available.

Lastly, in the sixth review, the authors pay particular attention to the benefits of plant-derived bioactives, namely ACDs/ACNs, in both the treatment and prevention of prostate cancer, through the analysis of preclinical evidence (in vitro and animal studies). To date, and despite cyanidin-3-O-glucoside as the most studied compound, ACDs/ACNs have excellent abilities, including inducing apoptosis, enhancing p21 expression, activating caspase-3, reducing Bcl-2, and increasing Bax expression. Nonetheless, as highlighted by the authors, most studies performed so far have addressed the activity of crude extracts, which are, thus, of utmost interest to deepen knowledge on the active phytochemicals responsible for the effects and to define the required doses for preventive or therapeutic approaches, along with their toxicological profiles.

Taken together, data published in this Special Issue underline the highly debatable and exciting field that is the study of the anticancer potential of phytochemicals. With plants being a rich source of active molecules, an intense investigation into this field has been conducted. However, less attention has been paid to the sustainable use of natural products. Indeed, despite the richness of natural matrices in secondary metabolites, some are present in vestigial amounts, and more importantly, some plants and natural products have a limited presence in nature, with some of them already being labeled “protected”. In this sense, one aim, more than ensuring a large battery of studies addressing the anticancer potential of naturally occurring bioactives, is to ensure their proper use. Thus, from the simplest screening studies up to the most detailed and complex experiments, including clinical trials, it is necessary to ensure that proper study protocols are launched; objective goals also need to be defined, with the ultimate aim of ensuring the translational approach of the findings stated. Something that is equally important to underline, and in a way also becomes accessible to all citizens, is to pay more attention to the chemical synthesis of the most renowned phytochemicals, not only to ensure that increasingly detailed studies are developed, including toxicological ones but also to ensure that they can be used at the right doses for preventive and therapeutic purposes without destroying nature and fostering environmental imbalance.
